# Evaluating time-based outcomes of a pharmacist–doctor collaborative discharge medication reconciliation model: an observational study

**DOI:** 10.1007/s11096-026-02118-y

**Published:** 2026-03-17

**Authors:** Suzanne Soudah, Matt Percival, Aaron Noble, John Dalziel, Catherine Edmunds, Kate Hill, H. Laetitia Hattingh

**Affiliations:** 1 Pharmacy, Gold Coast Hospital and Health Service, Southport, QLD 4215 Australia; 2 Allied Health Services, Chronic Disease and Post-Acute Programs, Gold Coast Hospital and Health Service, Southport, QLD 4215 Australia; 3 Allied Health Research, Gold Coast Hospital and Health Service, Southport, QLD 4215 Australia; 4 School of Pharmacy and Medical Sciences, Griffith University, Southport, QLD 4222 Australia; 5 School of Pharmacy and Pharmaceutical Sciences, The University of Queensland, Brisbane, QLD 4102 Australia

**Keywords:** Hospital discharge, Hospital pharmacist, Medication reconciliation, Pharmacist–doctor collaboration, Transition of care

## Abstract

**Introduction:**

Medication reconciliation at hospital discharge is essential to prevent medication discrepancies and ensure continuity of care. Competing clinical priorities often delay reconciliation, reducing discharge efficiency and increasing the risk of medication-related harm. Collaborative pharmacist–doctor models have potential to improve the quality and timeliness of discharge medication processes.

**Aim:**

To evaluate the impact of a pharmacist–doctor collaborative discharge medication reconciliation model on discharge timeliness, reconciliation quality, and pharmacist resource utilisation.

**Method:**

This observational study was conducted in a large tertiary hospital across two inpatient units over 12 weeks: six weeks usual care (1 September–12 October 2025) followed by 6 weeks intervention (13 October–28 November 2025). In the intervention phase, clinical pharmacists performed reconciliation planning, which involved preparing the draft discharge medication reconciliation plan for subsequent medical officer review and authorisation. Time-and-motion methodology captured discrete intervals across the discharge workflow. Quantile regression analysed time-based outcomes, and Poisson regression evaluated count-based outcomes including prescription adjustments. The primary outcome was time from discharge confirmation to patient departure; secondary outcomes included reconciliation completion rates, prescribing adjustments, and pharmacist workload.

**Results:**

A total of 116 patients were included (control n = 65; intervention n = 51). The collaborative model improved discharge efficiency, reducing the median time from discharge confirmation to leaving the ward by 78 min (*p* = 0.022). Time from decision to discharge to reconciliation completion was more than halved (30 vs 76 min, *p* < 0.001). Reconciliation completeness was significantly higher in the intervention group (90.2% vs 67.7%, *p* = 0.007), with fewer partial completions and no missing reconciliations. Analysis demonstrated earlier availability of discharge prescriptions (40 vs 80 min, *p* = 0.011) and shorter intervals between reconciliation completion and medication list preparation (14 vs 32 min, *p* = 0.008). Importantly, reconciliation planning by the pharmacist required a median of only 3 min per patient, confirming that improved timeliness required minimal additional pharmacist resourcing.

**Conclusion:**

A pharmacist–doctor collaborative discharge medication reconciliation model improved discharge efficiency and reconciliation accuracy without increasing pharmacist workload. These findings support broader implementation of collaborative models to enhance patient safety and hospital workflow performance. Further research should explore cost-effectiveness and patient-centred outcomes.

**Supplementary Information:**

The online version contains supplementary material available at 10.1007/s11096-026-02118-y.

## Impact statements

- Collaborative reconciliation models minimise duplication of effort and reduce discharge prescribing errors, supporting safer and more sustainable clinical workflows.
- The approach aligns with evolving healthcare policies promoting multidisciplinary models of care and optimised use of advanced pharmacy practice roles.
- Integrating pharmacists into discharge reconciliation reduces workflow bottlenecks and streamlines discharge pathways in busy inpatient units.

## Introduction

The hospital discharge process represents a critical transition in a patient’s healthcare journey as care shifts from an inpatient setting back to primary care providers [1, 2]. This period frequently involves medication changes, including discontinuing therapies, initiating new treatments, or adjusting doses [1, 3], representing a high-risk period for medication errors. Between 25 and 80% of patients are discharged with at least one medication discrepancy or inadequate communication of hospital-prescribed medications to the next phase of care [2, 4]. A 2018 systematic review reported that 21% of hospital readmissions were associated with medication related problems, with 69% considered preventable [5]. Medication errors remain the most preventable source of patient harm across hospitals, community settings, and residential aged care facilities [4, 6, 7].

Medication reconciliation is necessary at hospital discharge to reduce the opportunity for medication-related problems and improve patient safety [2, 4, 8, 9]. The World Health Organization (WHO) defines medication reconciliation as ‘the formal process in which health care professionals partner with patients to ensure accurate and complete medication information transfer at interfaces of care’ [2]. This process is a recognised safety intervention for preventing medication discrepancies and reducing the risk of patient harm during transitions of care [2, 4, 10–12], as it determines which medications are continued or stopped at the next phase [3]. Without reconciliation, discharge medication lists are highly susceptible to error [13]. Hospital pharmacists play a key role by completing timely reconciliation, providing patient and caregiver education, and preparing accurate discharge medication lists [14].

Preparing comprehensive discharge medication lists is complex and time-consuming [15], relying on the medical team to accurately complete discharge reconciliation promptly. This step is often delayed as doctors may need to prioritise competing clinical responsibilities [15, 16]. Junior doctors are responsible for a significant proportion of discharge medication orders, encompassing prescribing of discharge medications and completing the reconciliation process [17, 18]. Prescribing errors remain a concern in hospital settings where junior doctors are often responsible for entering of medication orders in medical records [17, 19–22]. Evidence indicates that junior doctors are approximately twice as likely as senior doctors to make prescribing errors [20].]. Hospital pharmacists therefore play a pivotal role in this context, identifying discrepancies, resolving errors and clarify prescriptions [22] as part of the multidisciplinary team collaborating to ensure safe and effective medication use [19].

In April 2025, amendments to Queensland’s Medicines and Poisons (Medicines) Regulation 2021 [23], enabled Collaborative Pharmacist Medication Prescribing (CPMP) within public and private healthcare facilities in Queensland, Australia [24]. Under CPMP, hospital pharmacists can undertake discharge medication reconciliation planning in collaboration with medical practitioners [25]. In this model of care, pharmacists prepare the reconciliation and discharge prescriptions, which are reviewed and co-signed by a doctor [16, 26]. Before completing the reconciliation, hospital pharmacists engage with patients early in their admission, for example in the emergency department or when they first arrive on the ward, to obtain an accurate history of medicines taken before admission, perform an admission medicines reconciliation, review documentation, and work with the treating team to finalise the discharge medication plan [26]. Unlike traditional workflows, where pharmacists conduct retrospective reviews following the prescriber's medication decisions, CPMP emphasises prospective intervention. While retrospective review often allows identification of prescribing errors, it may compromise discharge efficiency [27], by requiring repeated work, thereby delaying patient discharge and increasing workload [27].

Growing evidence highlights that pharmacist-led discharge reconciliation improves workflow efficiency [13], reduces medication discrepancies [1, 2, 9, 10, 12, 13, 18, 26–29], lowers adverse medication risk during care transitions [1, 12], prevents hospital readmissions [8, 14, 30] and enhances doctor and nurse satisfaction [26, 28, 31]. A recent national-level implementation study in Slovenia demonstrated how pharmacist-led medication reconciliation can be embedded across hospitals to strengthen medication safety and improve transitions of care at scale [32]. Building on these findings, this study sought to evaluate the impact of implementing a pharmacist-led discharge reconciliation model, focusing on discharge process timeframes and pharmacist resource utilisation.

### Aim

The aim of this study was to evaluate the impact of implementing a pharmacist–doctor collaborative discharge medication-reconciliation model on discharge-process efficiency. The study specifically sought to:

- Compare discharge process times between the intervention (CPMP model) and control (usual care) groups.
- Assess the effect of the intervention on pharmacist resource utilisation during discharge preparation.
- Explore differences in discharge medication reconciliation quality indicators, including prescription adjustments and completion rates.

This study was based on the hypotheses that implementing a pharmacist–doctor collaborative discharge medication reconciliation model would reduce discharge process times compared with usual care, decrease the frequency of prescription adjustments after initial reconciliation, and improve the overall quality of discharge medication reconciliation without increasing pharmacist workload.

## Method

This observational study evaluated the time-related impacts of a CPMP pharmacist-led discharge medication reconciliation model. This study was Phase 4 of a larger project: Phase 1 followed a consensus-building approach through nominal group technique methodology to rank discharge medication handover challenges and solutions [33], Phase 2 involved co-design of the multifaceted intervention aimed at improving medication discharge handover [34] and Phase 3 evaluated the effects of implementing the multifaceted intervention on discharge medication handover [35].

This evaluation was reported in accordance with the Strengthening the Reporting of Observational Studies in Epidemiology (STROBE) guidelines [36].

### Setting

This study was conducted at Gold Coast University Hospital (GCUH), a tertiary referral hospital with approximately 900 beds, operating under the Gold Coast Hospital and Health Service (GCHHS), which provides public healthcare to a population of over 680,000 residents across the Gold Coast region [37]. Data collection involved the Cardiology ward (24 beds) and a General Medicine ward known as the Specialist Medical Unit (27 beds). While each ward is supported by one dedicated pharmacist, the number of daily discharges varies according to patient acuity and hospital activity patterns.

### Current practice: control cohort

**Fig. 1:**
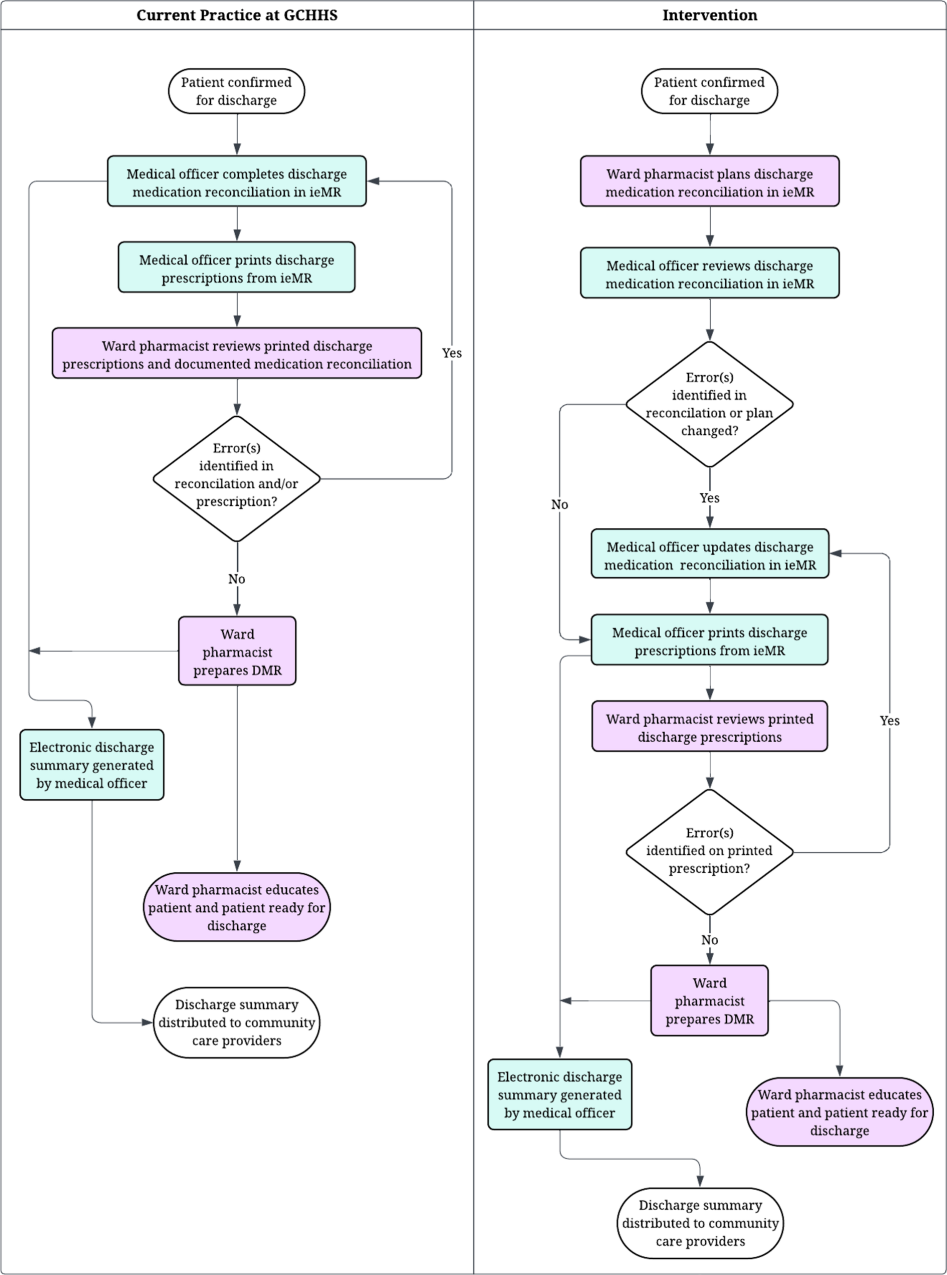
Current practice and proposed intervention workflows. *ieMR* = integrated electronic medical record; *DMR* = discharge medication record; *GCHHS* = Gold Coast Hospital and Health Service

Medical officers document patient information and medication plans through a Cerner®-based integrated electronic Medical Record (ieMR) system. Hospital pharmacists use the enterprise-wide Liaison Medication System (eLMS) to create patients’ Discharge Medication Records (DMRs), which incorporates medication changes and is provided to patients, included in discharge summaries to primary care doctors, and sent electronically to community pharmacies for dose administration aid support. Doctors are responsible for completing discharge reconciliation and issuing prescriptions at discharge initiation [38] (Fig. 1).

### Intervention: intervention cohort

Hospital pharmacists planned discharge medication reconciliation in collaboration with medical officers (interns, registrars, trainees or consultants). The intervention was implemented on weekdays in a staggered schedule: October 1–November 14, 2025, in one Cardiology ward and October 13–November 28, 2025, in the Specialist Medical Unit. This approach supported staff engagement prior to commencement. Two pharmacists assigned to the units completed training on the workflow and documentation requirements within ieMR [34]. The intervention workflow involved pharmacists planning the reconciliation and preparing draft prescriptions for subsequent medical review and approval (Fig. 1).

### Participants

Inclusion criteria:

- Admission > 24 h
- Discharge under a medical team participating in the intervention, and
- A DMR completed by a hospital pharmacist prior to discharge.

### Data collection

#### Audit data

The aim was to include approximately 50 patient discharges per each of the control and intervention cohorts [39]. The project pharmacist (SS) extracted retrospective data for both intervention and control cohorts from ieMR, eLMS, and My Health Record:

- Age and gender,
- Discharge destination,
- Pharmacist admission history status at time of confirmed discharge,
- Discharge reconciliation status,
- Number of DMR items, and
- Number of medication changes during admission (ceased, new, changed, with-held)

#### Time-and-motion data

Time-and-motion methodology was used to quantify the duration of discrete discharge workflow steps for both cohorts over 12 weeks (six weeks control followed by six weeks intervention). This methodology involves the prospective, real-time recording of discrete workflow events to quantify the duration of specific tasks within a clinical process [40]. In this study, hospital pharmacists recorded predefined timestamps in a structured Excel spreadsheet, using the hospital’s standardised electronic time display to ensure consistency across observers. For each patient, pharmacists documented the exact clock time at which the following events occurred:

- Discharge confirmation,
- Discharge reconciliation completion,
- Receipt of discharge prescriptions,
- DMR authorisation, and
- Patient departure from the ward.

The primary endpoint was defined as the patient leaving the ward, as this is when the inpatient bed becomes available. Any time spent afterward, such as waiting in the transit lounge for medications or transport, was not included because it no longer affects bed availability or patient flow. Times were entered immediately after each event occurred to minimise recall bias. To ensure consistency in recording, the project pharmacist conducted observational audits on 20% of the hospital pharmacists’ activities. Discrepancies in time logging between the hospital pharmacists and the project pharmacist exceeding 10% was set to prompt additional training to reinforce accurate data collection practices.

Additional variables recorded included:

- Frequency of doctor-initiated reconciliation adjustments,
- Pharmacist requests for clarification, and
- Discharge delays relative to the confirmed discharge date.

To preserve the validity of time-based analyses, data exclusions were applied at the variable level rather than the patient level. Observations were removed only for specific time-related variables when documentation was incomplete or inconsistent, while patients were retained for other analyses where data were available. Exclusion criteria for time variables included:

- Discharge reconciliation completed on a date different from the confirmed discharge date,
- No discharge reconciliation recorded,
- DMR authorised on a different date to the confirmed discharge date,
- Discharge delayed where the leave date did not match the confirmed discharge date,
- Missing documentation of time prescription received and
- Instances where no prescriptions were required.

### Statistical analysis

All analyses were performed using Stata version 19 (StataCorp LLC, College Station, TX, 2025). Continuous variables were assessed for distributional characteristics. Parametric continuous data were reported as means with standard deviations (SD), and non-parametric continuous data as medians with interquartile ranges (IQR). Categorical variables were summarised as frequencies and percentages. Between-group comparisons were conducted using independent-samples *t*-tests for parametric data and the Mann–Whitney test for non-parametric data, while categorical variables were analysed using Pearson’s chi-square or Fisher’s exact test.

Discharge process times were positively skewed; therefore, quantile regression was used to compare medians and estimate 95% confidence intervals (CI) for differences between groups. This approach was chosen for its robustness to non-normal distributions and its ability to provide interpretable estimates of median differences. For selected outcomes involving count data, Poisson regression was applied to estimate incidence rate ratios (IRRs) with 95% CIs, complementing non-parametric tests by quantifying the magnitude of differences between groups.

### Ethics approval

This study was approved by the Gold Coast Hospital and Health Service (GCHHS) Human Research Ethics Committee (Ref No. HREC/2023/QGC/94932).

## Results

A total of 116 patients were included in the analysis: 65 in the control group (32 cardiology, 33 general medicine) and 51 in the intervention group (37 cardiology, 14 general medicine) (Table 1). Baseline demographic and clinical characteristics were similar between groups. Observational audits showed similar time logging between the hospital pharmacists and the project pharmacist.

**Table 1:** Intervention and control patients’ baseline demographic characteristics

Variable	Control, *n* = 65	Intervention, *n* = 51	*P* value
*Ward, n (%)*			
Cardiology unit	32 (49.2)	37 (72.5)	0.011*
General Medicine Specialist Medical unit	33 (50.8)	14 (27.5)	
*Age, years*			
Mean (SD)	71.1 (17.0)	66.7 (14.8)	0.146
*Gender, n (%)*			
Female	28 (43.1)	18 (35.3)	0.395
Male	37 (56.9)	33 (64.7)	
*Discharge Destination, n (%)*			
Home	45 (69.2)	44 (86.3)	0.120
Existing RACF	6 (9.2)	2 (3.9)	
New RACF	4 (6.2)	3 (5.9)	
Other^a^	10 (15.4)	2 (3.9)	

^a^Transitional care program; *SD* = standard deviation; *RACF* = residential aged care facility. Percentages are column-based (within group). * The p-value assesses whether the distribution of participants by ward (Cardiology vs General Medicine) differs between control and intervention groups

### Comparison of discharge medication processes between control and intervention cohorts

Medication changes during admission and at discharge were similar between groups (Table 2). The median number of medications on the DMR was 13 (IQR 7, 16) in the control group and 12 (IQR 8, 14) in the intervention group. Similarly, there were no differences in the number of new medications added, medications changed, medications with-held or ceased during hospitalisation. Tapering or dose titration plans were documented on the discharge medication record (DMR) for four patients in the control group (8%) and seven patients in the intervention group (14%)**.**

**Table 2:** Medication changes during admission and at discharge

Number of:	Control, *n* = 65	Intervention, *n* = 51	*P* value
Medications on DMR, median [IQR]	13 [7, 16]	12 [8, 14]	0.848
New medications added in hospital, median [IQR]	3 [1, 5]	2 [1, 4]	0.443
Medications changes made in hospital, median [IQR]	0 [0, 1]	1 [0, 1]	0.697
Medications ceased in hospital, median [IQR]	1 [0, 2]	0 [0, 2]	0.672
Medications withheld in hospital, median [IQR]	0 [0, 0]	0 [0, 0]	0.605

*IQR* = interquartile range; *DMR* = discharge medication record

Hospital discharge summary details are summarised in Table 3. Pharmacist admission history was completed prior to confirmed discharge for 84.3% of intervention patients compared with 75.4% of control patients (*p* = 0.239). Medication reconciliation at discharge was more frequently completed in the intervention group (90.2% vs. 67.7%), with fewer partial completions (9.8% vs. 26.1%) (*p* = 0.007). The median number of prescriptions generated for discharge was similar (4 [IQR 2, 5] intervention vs. 3 [IQR 2, 5] control, *p* = 0.551). The number of discharge reconciliation adjustments by the doctors was 0 [IQR 0–1] for intervention vs 1 [IQR 0–2] for control groups (*p* < 0.001).

**Table 3:** Hospital discharge summary details

Variable	Control, *n* = 65	Intervention, *n* = 51	*P* value
*Pharmacist admission history completed prior to confirmed discharge, n (%)*			
No	16 (24.6)	8 (15.7)	0.239
Yes	49 (75.4)	43 (84.3)	
*Medication reconciliation completed at discharge, n (%)*			
No	4 (6.2)	0	0.007
Yes	44 (67.7)	46 (90.2)	
Partially	17 (26.1)	5 (9.8)	
*Number of prescriptions generated for discharge*			
Median [IQR]	3 [2, 5]	4 [2, 5]	0.551
*Number of times discharge reconciliation adjusted by medical officer*			
Median [IQR]	1 [0, 2]	0 [0, 1]*	< 0.001

*After discharge reconciliation plan completed by pharmacist. *IQR* = interquartile range. Percentages are column-based (within group)

Patients who had a pharmacist-completed medication admission history achieved significantly faster discharge reconciliation, with a median time of 45 min from confirmed discharge compared with 80 min in those without this history, representing an average 35-min reduction (*p* = 0.032; 95% CI − 66.66 to − 3.34).

### Comparison of discharge time components between control and intervention cohorts

Time taken for discharge processes was consistently shorter in the intervention group compared with the control group (Table 4). The median time from discharge confirmation to completion of medication reconciliation by the doctor was significantly reduced in the intervention group (30 min vs 76 min; *p* < 0.001). Similarly, the time to receive discharge prescriptions was shorter (40 min vs 80 min; *p* = 0.011). Completion of the DMR occurred earlier in the intervention group (71 min vs 114 min; *p* = 0.053). Reductions were observed for the interval from reconciliation completion by the doctors to DMR completion (14 min vs 32 min; *p* = 0.008). In contrast, no difference was observed for the interval between receiving prescriptions and DMR completion (15.5 min vs 15 min; *p* = 0.649).

**Table 4:** Time taken for discharge processes

Activity	Control, *n* = 65	Intervention, *n* = 51	Median difference (95% CI)	*P* value
	Median (minutes)	Median (minutes)		
Time discharge confirmed to reconciliation completed^a^	76	30	− 46 (− 69.8, − 22.2)	< 0.001
Time discharge confirmed to scripts received^b^	80	40	− 40 (− 70.5, − 9.5)	0.011
Time discharge confirmed to DMR completion	114	71	− 41 (− 82.6, + 0.6)	0.053
Time discharge reconciliation completed^a^ to scripts received ^b^	8	5	− 3 (− 7.4, + 1.4)	0.178
Time discharge reconciliation completed^a^ to DMR completion	32	14	− 19 (− 32.9, − 5.1)	0.008
Time scripts received^b^ to DMR completion	15	15	+ 2 (− 6.7, + 10.7)	0.649
Time discharge confirmed to patient leaving ward	226	143	− 78 (− 144.8, − 11.2)	0.022

^a^by doctor; ^b^by pharmacist. Median differences and 95% CIs estimated using quantile regression (τ = 0.5). *DMR* = discharge medication record

Overall, patients in the intervention group left the ward a median of 78 min earlier than those in the control group (143 min vs 226 min; p = 0.022). Both groups exhibited bimodal distributions of discharge timing. 14 patients (14%) had a delayed discharge, with equal numbers in the control and intervention groups (7 each). A delay was defined as failure to leave the ward on the same day as discharge reconciliation.

### Intervention time components

Time intervals specific to the intervention are shown in Table 5. Pharmacists commenced reconciliation planning a median of eight minutes after discharge confirmation (95% CI 4.0–12.0) and required a median of three minutes to complete the plan (95% CI 2.2–3.8). The interval from pharmacist planning to medical officer completion was 17 min (95% CI 6.0–28.0), and the time from planning to receiving discharge prescriptions was 23 min (95% CI 4.7–41.3). For 15 of 51 patients (29%) in the intervention group, pharmacists completed the discharge reconciliation plan without direct input from medical officers, typically documentation was sufficiently detailed, or no medication changes had taken place during the admission. Conversely, in five patients (10%), the discharge reconciliation was initially commenced by the doctor. However, due to identified errors and discrepancies, the ward pharmacist repeated the reconciliation before finalisation by the medical team. Out of 51 reconciliation plans by the pharmacist, 14 (27%) required adjustment by the doctor, with 10/14 (71%) attributable to unexpected last-minute changes in the treatment plan.

**Table 5:** Time taken for discharge processes specific to intervention

Activity:	Intervention	
	Median (mins)	95% CI
Time discharge confirmed to reconciliation planned ^b^	8	4.0, 12.0
Time to complete discharge reconciliation plan^b^	3	2.2, 3.8
Time discharge reconciliation planned^b^ to reconciliation completed^a^	17	6.0, 28.0
Time discharge reconciliation planned^b^ to scripts received^b^	23	4.7, 41.3

^a^ by doctor; ^b^by pharmacist. 95% CIs estimated using quantile regression (τ = 0.5)

## Discussion

This study evaluated the impact of a pharmacist–doctor collaborative discharge medication reconciliation model on discharge efficiency and pharmacist resource utilisation. The intervention substantially reduced time from discharge confirmation to leaving the ward, with patients departing a median of 78 min earlier than controls. Although both groups exhibited bimodal discharge patterns, the intervention shifted the distribution toward earlier discharge, reducing delays. Other key time intervals also improved, including discharge confirmation to reconciliation completion (30 min vs 76 min; *p* < 0.001) and reconciliation completion to DMR completion (14 min vs 32 min; *p* = 0.008). Pharmacist workload was unaffected, with reconciliation planning requiring a median of three minutes per patient. As expected, intervals recorded after receiving prescriptions did not differ, as these steps were not targeted by the model. Collectively, these findings demonstrate that pharmacist-led discharge reconciliation improves discharge timeliness, enhances accuracy, with minimal impact on pharmacist resource utilisation. The distribution of patients by ward differed between groups (Table 1), with a greater proportion of Cardiology patients in the intervention cohort. As workflow patterns, prescribing practices and discharge readiness can vary between clinical units, this imbalance may have contributed to differences in time-based outcomes. Although our primary analyses were unadjusted, this ward variation should be considered when interpreting the magnitude of the observed effects.

Timely discharges are critical for maintaining bed availability, patient flow, and throughput in high-turnover wards operating at near-capacity [28, 29, 37]. A common barrier to timely discharge is delayed reconciliation by junior doctors [15, 16, 41], which disrupts pharmacists’ ability to prepare discharge medication lists and verify prescriptions [16]. By enabling pharmacists to initiate reconciliation earlier, this model reduced dependency on doctors’ availability and alleviated workflow bottlenecks that commonly prolong discharge [28, 29]. These earlier completions translated directly into earlier patient departures, demonstrating the model’s potential to ease bed block and improve operational efficiency.

The improvement in reconciliation completeness (90.2% vs 67.7%; p = 0.007) and the reduction in doctor adjustments (p < 0.001) reinforce evidence that pharmacist led reconciliation enhances medication accuracy. These results align with studies demonstrating reductions in clinically significant discharge medication errors from 61.9 to 9.3% [42] and a 58% decrease in discharge discrepancies when pharmacists lead reconciliation [30]. Systematic reviews further confirm that pharmacist involvement across transitions of care improves medication list accuracy and reduces adverse drug events, when combined with counselling and follow-up [8, 14]. The present findings therefore support the established role of pharmacists in improving medication safety at discharge.

Beyond safety benefits, collaborative pharmacist–doctor models have been associated with shorter hospital stays [28], improved staff satisfaction [18, 26, 28, 43, 44], fewer readmissions and enhances continuity of care [8, 14, 45]. Concerns that these models limit junior doctors’ learning opportunities are not supported by evidence. Doctors retain responsibility for reviewing and authorising prescriptions [26], preserving ownership of clinical decision-making. Collaboration fosters interprofessional learning, offering real-time feedback from pharmacists that improves prescribing accuracy and confidence [21, 22, 31, 44]. By enabling pharmacists to undertake these components of the workflow, the model frees junior doctors to concentrate on other clinical activities such as patient assessment and treatment planning [29]. Rather than diminishing educational exposure, the model fosters a supportive learning environment that helps prevent errors and reinforces prescribing skills [18, 30, 42]. Although some reviews emphasise the need for standardised reconciliation processes and economic evaluations [11, 12], available analyses suggest only modest additional costs, with potential savings from preventing adverse drug events and readmissions far outweighing the expense [46].

The outcomes of this study reinforce the need for institutional policies that embed pharmacists as core partners in discharge planning. This aligns with WHO recommendations for improving medication safety during care transitions [2] and with the Australian Commission on Safety and Quality in Health Care’s stewardship framework [9]. The model is particularly relevant in the context of Queensland’s recent legislative changes enabling CPMP [24, 25], providing a practical approach to optimise discharge processes under contemporary regulatory arrangements. Further support for this approach comes from recent international work, including a nationwide initiative in Slovenia that led to notable gains in discharge accuracy and timeliness [32]. Furthermore, as highlighted in this study, timely completion of pharmacist admission histories provides a critical foundation for efficient discharge planning by ensuring early verification of medication information, thereby preventing delays that may otherwise increase discharge medicine list preparation time. These findings are similar to a study that showed approximately one minute per undocumented medication is saved at the time of discharge with pharmacist admission histories done earlier in patients’ hospital admission [15].

This study’s strengths include implementation in a real-world tertiary hospital and the involvement of a dedicated research pharmacist, ensuring consistent documentation. Nevertheless, limitations should be acknowledged. The single-site design, small sample size and short study duration may limit generalisability. Patient-centred outcomes, such as satisfaction, adverse drug events, readmissions and medication adherence were not assessed, and no economic analysis was performed. Junior doctor rotation created variability in exposure to the model, and bed block in the General Medical unit reduced the number of eligible discharges. Recruitment imbalance between units reflects pragmatic operational constraints rather than systematic bias. Finally, while findings may have limited applicability in countries with different models of care, outcomes are likely transferable to other jurisdictions with similar hospital workflows.

A further consideration is the resource context in which this model was implemented. The study site is already well supported by pharmacists, which enabled the CPMP pharmacist-led discharge reconciliation model to be introduced without additional staffing. This may not be the case in settings with lower baseline pharmacist capacity, where additional investment may be required to achieve comparable outcomes. Nonetheless, the efficiencies demonstrated suggest that the model may offer sufficient operational and clinical value to justify such investment in resource-constrained environments.

## Conclusion

This observational study demonstrates that a pharmacist–doctor collaborative discharge medication reconciliation model can significantly improve discharge efficiency and reconciliation accuracy without increasing pharmacist workload. The model addresses key workflow challenges and strengthens continuity of care. These findings support broader adoption of collaborative approaches to become standard practice in high-turnover inpatient settings. Future research should incorporate patient-centred outcomes, clinical safety endpoints and economic analyses to inform scalability and long-term sustainability across diverse healthcare settings.

## Supplementary Information

Below is the link to the electronic supplementary material.

Supplementary file1 (DOCX 39 KB)

## Data Availability

De-identified datasets used and/or analysed during the current study are available from the corresponding author on reasonable request.
